# An updated review of the immunological mechanisms of keloid scars

**DOI:** 10.3389/fimmu.2023.1117630

**Published:** 2023-03-22

**Authors:** Chih-Chun Lee, Chia-Hsuan Tsai, Chih-Hao Chen, Yuan-Chieh Yeh, Wen-Hung Chung, Chun-Bing Chen

**Affiliations:** ^1^ 1 Department of Medical Education, Chang Gung Memorial Hospital, Keelung, Taiwan; ^2^ Department of Plastic and Reconstructive Surgery, Chang Gung Memorial Hospital, Keelung, Taiwan; ^3^ College of Medicine, Chang Gung University, Taoyuan, Taiwan; ^4^ Department of Traditional Chinese Medicine, Chang Gung Memorial Hospital, Keelung, Taiwan; ^5^ Program in Molecular Medicine, College of Life Sciences, National Yang Ming Chiao Tung University, Taipei, Taiwan; ^6^ Drug Hypersensitivity Clinical and Research Center, Department of Dermatology, Chang Gung Memorial Hospital, Linkou, Taiwan; ^7^ Drug Hypersensitivity Clinical and Research Center, Department of Dermatology, Chang Gung Memorial Hospital, Taipei, Taiwan; ^8^ Drug Hypersensitivity Clinical and Research Center, Department of Dermatology, Chang Gung Memorial Hospital, Keelung, Taiwan; ^9^ Cancer Vaccine and Immune Cell Therapy Core Laboratory, Chang Gung Memorial Hospital, Linkou, Taiwan; ^10^ Chang Gung Immunology Consortium, Chang Gung Memorial Hospital and Chang Gung University, Linkou, Taiwan; ^11^ Department of Dermatology, Xiamen Chang Gung Hospital, Xiamen, China; ^12^ Xiamen Chang Gung Allergology Consortium, Xiamen Chang Gung Hospital, Xiamen, China; ^13^ Whole-Genome Research Core Laboratory of Human Diseases, Chang Gung Memorial Hospital, Keelung, Taiwan; ^14^ Immune-Oncology Center of Excellence, Chang Gung Memorial Hospital, Linkou, Taiwan; ^15^ Graduate Institute of Clinical Medical Sciences, College of Medicine, Chang Gung University, Taoyuan, Taiwan; ^16^ Genomic Medicine Core Laboratory, Chang Gung Memorial Hospital, Linkou, Taiwan; ^17^ School of Medicine, National Tsing Hua University, Hsinchu, Taiwan

**Keywords:** keloid, scar, immunity, macrophages, T lymphocytes, cytokines, signal transduction

## Abstract

Keloid is a type of disfiguring pathological scarring unique to human skin. The disorder is characterized by excessive collagen deposition. Immune cell infiltration is a hallmark of both normal and pathological tissue repair. However, the immunopathological mechanisms of keloid remain unclear. Recent studies have uncovered the pivotal role of both innate and adaptive immunity in modulating the aberrant behavior of keloid fibroblasts. Several novel therapeutics attempting to restore regulation of the immune microenvironment have shown variable efficacy. We review the current understanding of keloid immunopathogenesis and highlight the potential roles of immune pathway-specific therapeutics.

## 1 Introduction

Keloid is a type of pathological scarring unique to human skin. The disorder is characterized by dysregulated fibroproliferation with excessive production of extracellular matrix (ECM) and extension beyond the initial wound (1). Keloid scars are often disfiguring, profoundly impair the quality of life and cause immense physical and mental distress of affected individuals, especially in those with symptomatic (pruritic, painful) and/or hyperpigmented scars (2–4). Limited epidemiological data suggested a female predominance, and a higher prevalence among people of darker skin complexion, such as those of African and Asian descents (5, 6). The prevalence of excessive scarring in Black, Asians and Caucasians was recently reported at 2.4%, 1.1% and 0.4%, respectively (6). Association between excessive scarring and other systemic conditions including hypertension (7–9), vitamin D deficiency (10, 11), and atopic dermatitis (12, 13) has been suggested. A recent cohort of the UK biobank found atopic dermatitis significantly associated with excessive scarring across ethnic groups (6). Hypertension in Blacks and vitamin D deficiency in Asians also showed significant association with keloid formation (6).

In practice, several preventive and therapeutic therapies are used to manage keloids. Application of silicone gel sheets, topical corticosteroids, and intralesional corticosteroids are frequently utilized in individuals with a history of excessive scarring after trauma or surgeries (14). For established keloids, nonsurgical management commonly involves intralesional corticosteroids (e.g., triamcinolone acetonide) (5). Laser-assisted topical steroid application is a novel alternative with better reported aesthetic outcome (15). Intralesional injection of botulinum toxin A, 5-fluorouracil, verapamil, bleomycin, and interferon (IFN)-α2b are less common measures with varying efficacy (16, 17). Other methods include laser therapy (18), and intralesional cryosurgery (19). Monotherapy with radiation is less preferred due to the requirement of large radiation doses (14). Successful surgical management of keloids hinges on the ability to minimize dermal tension (20). Body site-specific techniques have been proposed (14). The high postsurgical recurrence rate can be ameliorated with adjunctive radiation and/or local corticosteroids (14). There are also anecdotal reports with tissue-engineered allografts (21) and platelet-rich plasma (22). The associated adverse effects of established therapies could be significant, especially with long-term or repeated treatment. Intralesional corticosteroids, one of the most frequent methods in both prophylactic and therapeutic management of keloids, is associated with skin hypo-/hyper-pigmentation, atrophy, and telangiectasia.

The pathogenesis of the exuberant scarring remains incompletely understood. No single determining pathway has been identified. Instead, roles of several transcription factors, growth factors, cytokines, ECM proteins, and their associated regulators/effectors have been implicated in experimental studies. The dysregulated molecular profile causes imbalance within and across stages of tissue repair. Wound healing consists of an overlapping sequence of hemostasis, inflammation, proliferation and re-epithelization, and remodelling (23, 24). At the inflammation stage, the innate immune system is activated in response to the damage-associate molecular patterns (DAMPs) and other danger signals (23, 24). Cell debris are removed *via* phagocytosis of neutrophils (23, 24). Macrophages are later recruited. In addition to phagocytosis, macrophages play an important role in the resolution of inflammation, setting the stage for proliferation (23, 24). The proliferation phase is characterized by migration of keratinocytes, angiogenesis, and formation of granulation tissue (23, 24). Remodelling ensues with replacement of collagen III with collagen I and regression of blood vessels (23, 24). Across the stages, there is a complex interplay between immune cells and fibroblasts. Moreover, the outcome of subsequent stages is closely associated with the integrity and functionality of prior events (23). Hence, excessive scarring could arise as primary dysfunction of the remodelling phase or secondary to an exaggerated inflammatory response (23, 25).

The etiology of keloids is likely multifactorial and hinges on a constellation of factors, including genetic predisposition (26–34), inflammation (35–39), mechanical stress (40–43), tissue hypoxia (44–48), delayed-type hypersensitivity (49), and metabolic dysfunction (50, 51). Familial cases of autosomal dominant inheritance with incomplete clinical penetrance and variable expression have been described (52–54). Several immune pathway-associated susceptible genotypes have been identified, including polymorphisms of interleukin (IL)-6 and transforming growth factor (TGF)-β receptors (26–28, 55–57). Moreover, immune cell infiltration is a hallmark of keloid tissue. Preferential recruitment of immune cells modulates the process of skin repair *via* interaction with keloid fibroblasts (58, 59). Since the 1970s, the immunological aspect of keloid formation has been proposed (60–63), and a potential role for autoimmunity was frequently evoked in early reports (60, 63). With the advent of novel technologies and laboratory methods, the interest in the immunological landscape of keloid formation has led to vigorous investigations over the past two decades.

The reticular dermis has been proposed as the main locale of chronic inflammation underscoring the formation of keloid scars with upregulation of various proinflammatory cytokines, including IL-1α, IL-1β, IL-6, and tumor necrosis factor (TNF)-α (35). Interestingly, there appeared to be a concomitant excess of regulatory cell types and cytokines (64, 65). Study of keloid histology demonstrated altered expression of ECM molecules with increased type I/III collagen ratio, and a hypercellular dermis with increased numbers of fibroblasts, mast cells and macrophages, as well as varying presence of lymphocytes (1, 36, 64, 66–75). Keloid tissue also harbored a higher percentage of mesenchymal stem cells, and the amount of which was found to be correlated with disease recurrence (76). The role of myofibroblasts is less defined. A recent report found that myofibroblasts, a key feature of cultured fibroblasts in several reports, are not characteristic of keloid lesion *in vivo* (77). The concept of keloid microenvironment has been frequently evoked to describe the complex cellular and molecular interplay that gives rise to and sustains keloidogenesis. Recent technologies, such as identification of differentially expressed genes *via* examination of RNA sequencing data sets (74, 75, 78–80), have led to more extensive analysis of keloid tissue. A skewed T helper (Th) 2 phenotype was recently characterized (81–85), along with a potential co-susceptibility of keloids and atopic dermatitis (6, 12, 86). Moreover, even in the absence of comorbid atopic dermatitis, both lesional and non-lesional skin of patients with chronic keloids exhibit Th2 predominance (81). These features of heightened immune response were validated in a recent transcriptomic study, in which a globally elevated expression of several immune pathways over the entire integument of keloid patients was seen, especially the Th2 and Janus kinase (JAK) 3 pathways. Increased expression of T cell, regulatory T cell (Treg), and dendritic cell (DC) markers was also observed, along with the expression of the innate, Th1- and Th17/Th22-signaling pathways (85). The change in cellular composition and function is accompanied by increased levels of IL-6, IL-10, IL-17, TGF-β, and TNF (38, 39, 48, 78, 85, 87–94). Increase in IL-4, IL-13, IL-18, granulocyte colony-stimulating factors, and granulocyte-monocyte colony-stimulating factors, were also observed (39, 82–85, 87, 88). On the other hand, reduced expression of potential anti-inflammatory mediators, such as IL-34 and IL-37, has been reported (85, 87, 95, 96). Single-cell RNA sequencing and spatial transcriptomics (72, 97–99), as well as epigenetics (34, 100) are emerging fields utilized to reveal potential pathophysiological features of keloids. One single-cell RNA sequencing study identified a distinct macrophage-centered communication regulatory network that may favor transition and proliferation of M2 macrophages (72). In addition to the local characteristics, corresponding abnormalities in the cytokine profile have been identified in the peripheral blood of keloid patients (39). Serum soluble human leukocyte antigen-E (sHLA-E) was recently identified as a potential biomarker of keloid occurrence and recurrence (101). Furthermore, aberrant immune cell composition and activity are increasingly recognized in the non-lesional skin of keloid patients (81, 82, 85). Reports on the involvement of humoral immunity were less consistent. Anti-hnRNPA2B1, an autoantibody against RNA-associated proteins, was found to be significantly elevated in the serum of keloid patients (73). The same study also showed deposition of immunoglobulins (IgA, IgM) and complements (IgA, IgM, C3 and C1q) *via* immunofluorescence in keloid skin tissue (73). These findings suggest a systemic pathological process underscoring the development of keloids, such that the risk-benefit of repeated local therapy for susceptible individuals is called into question. Further studies are required to elucidate the origin of the keloid-prone immunological signatures.

In this review, current understanding of keloid immunopathogenesis is discussed, with highlights of potential pathway-targeted therapeutics.

## 2 The roles of immune cells in keloid formation

### 2.1 Mast cells as profibrotic mediators

**Figure 1:**
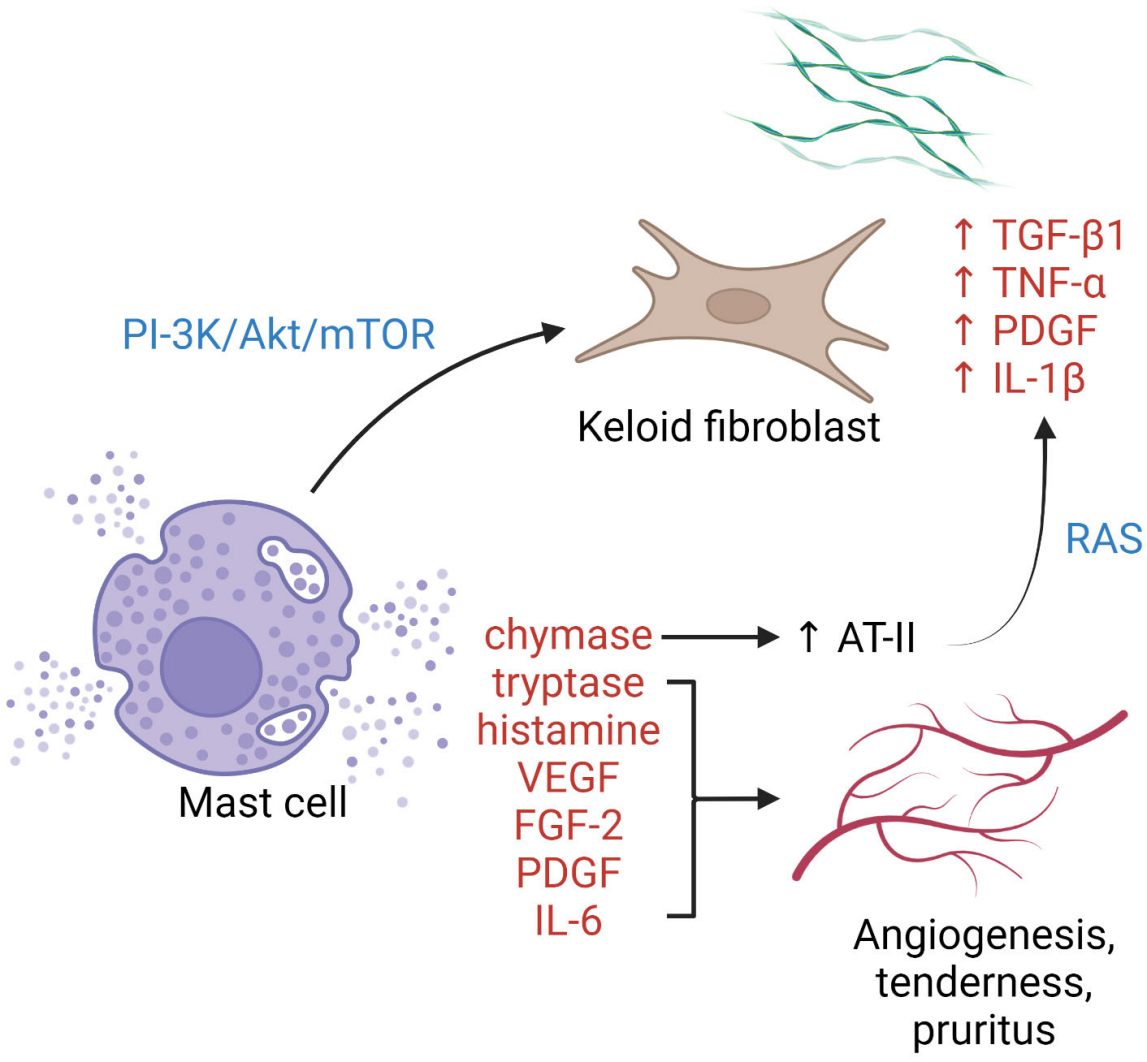
The role of mast cells in keloid pathogenesis. Degranulated mast cells crosstalk with activated keloid fibroblasts *via* the PI3K/Akt/mTOR pathway, leading to more collagen synthesis. Enzymes, growth factors, and cytokines released upon mast cell degranulation contribute to activation of the RAS, upregulation of keloid fibroblasts, angiogenesis, and cutaneous symptoms. *AT-II*, angiotensin II; *FGF-2*, fibroblast growth factor 2; *IL*, interleukin; *PDGF*, platelet-derived growth factor; *PI3K/Akt/mTOR*, phosphatidylinositol-3-kinase/Akt/mammalian target of rapamycin pathway; *RAS*, renin–angiotensin system; *TGF-β*, transforming growth factor-β; *TNF-α*, tumor necrosis factor-α; *VEGF*, vascular endothelial growth factor.

Mast cells cluster in tissue exposed to the external environment. In human skin, mature mast cells are abundant near the vasculature, lymphatics, nerves, and fibroblasts, and play a crucial role in wound healing by initiating inflammation, facilitating re-epithelialization, and inducing angiogenesis (68). It has been postulated that mast cells contribute to profibrotic chronic inflammation as well as to the common symptoms (pruritus and erythema) associated with keloid scars (**Figure 1**). Silicone gel sheeting has been shown to reduce mast cell infiltration in keloid lesions and thus provide symptomatic relief (102).

Increased intralesional and perilesional mast cells can be observed in keloid tissue, both perivascularly and within abnormal collagen bundles (36, 103). Degranulated mast cells are frequently seen in contact with active fibroblasts, indicating the presence of cell–cell interaction (67, 103). Attenuation of such cellular crosstalk has been achieved by blockading the phosphatidylinositol 3-kinase (PI3K)/Akt/mammalian target of rapamycin (mTOR) pathway using green tea extract (polyphenol EGCG), with a corresponding reduction in type I collagen production (104). Several pro-angiogenic factors are released by mast cells, including vascular endothelial growth factor (VEGF), fibroblast growth factor-2, platelet-derived growth factor, IL-6, tryptase, and chymase. Tryptase, a serine protease, is one of the most potent inducers of tissue angiogenesis. Tryptase-positive mast cell density and keloid angiogenesis are positively correlated (105). The use of transdermal tryptase inhibitors for hypertrophic scars and keloids has been described with symptomatic benefit (105). Mast cell chymase expression and activity are heightened in keloid tissue. The enzyme is profibrotic and stimulates fibroblast proliferation and collagen synthesis *via* the TGF-β1/Smad signaling pathway (106). Mast cell-derived chymase enhances angiotensin II expression, leading to local activation of the renin-angiotensin system and upregulation of TGF-β1, TNF-α, platelet-derived growth factor, and IL-1β in keloid fibroblasts (107). Chymase inhibitors have been shown to possess antifibrotic property in skin (108), cardiovascular system (109), and liver (110) in animal models. Other means of mast cell antagonization, e.g., with mast cell stabilizers (111) or tyrosine kinase inhibitors (112), have not been tested in keloids.

### 2.2 Macrophage polarization and chronic inflammation

**Figure 2:**
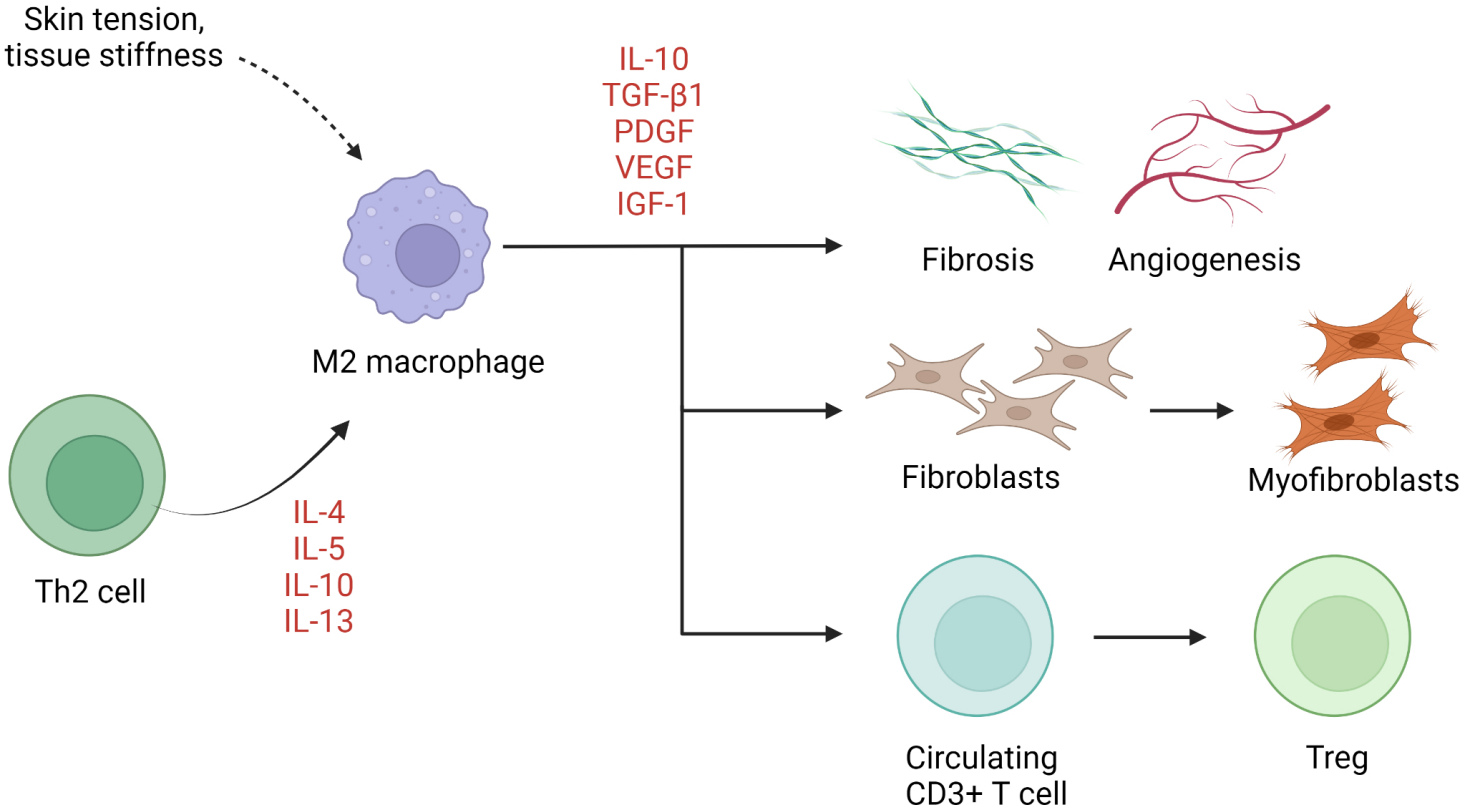
The role of macrophages in keloid pathogenesis. M2 macrophages predominate in keloids, resulting in activation of keloid fibroblasts, Treg differentiation, and fibrosis and angiogenesis. *IGF-1*, insulin-like growth factor 1; *IL*, interleukin; *PDGF*, platelet-derived growth factor; *TGF-β1*, transforming growth factor β1; *Th*, helper T cells; *Treg*, regulatory T cell; *VEGF*, vascular endothelial growth factor.

M1 (classically activated, CD68-positive) and M2 (alternatively activated, CD163-positive) are two well-established macrophage subgroups. The two phenotypes possess opposing properties, with the former exerting a pro-inflammatory effect and the latter an anti-inflammatory effect (113). An imbalance between M1 and M2 macrophages has been described in several chronic inflammatory conditions such as rheumatoid arthritis (114). Normal wound healing is characterized by an orchestrated transition from M1-predominant early inflammatory stages to M2-predominant restitution (115). Dysregulation of this process leads to either prolonged inflammation with delayed wound closure or increased scarring. M2 macrophages are disproportionally elevated in keloid lesions (69–72, 74), in part due to local enrichment of Th2 cytokines. Although not yet verified, M2 dominance has also been linked to macrophage sensitivity to mechanical signals, including skin tension and stiffness (42). M2 macrophages initiate wound closure *via* secretion of TGF-β1, a potent inducer of both fibroblast proliferation and their differentiation into myofibroblasts (115). Moreover, M2 macrophages induce transcription factor forkhead box P3 (FOXP3) expression in circulating CD3+ T cells, contributing to the formation of Tregs (64) (**Figure 2**). Interestingly, while M2 predominance is clearly present, expression of both M1 (inducible nitric oxide synthase [iNOS], IL-12)- and M2 (IL-10, TGF-β)-associated genes and proteins is enhanced keloid lesions compared to normal skin (64).

### 2.3 Tregs-derived TGF-β1 and collagen expression

**Figure 3:**
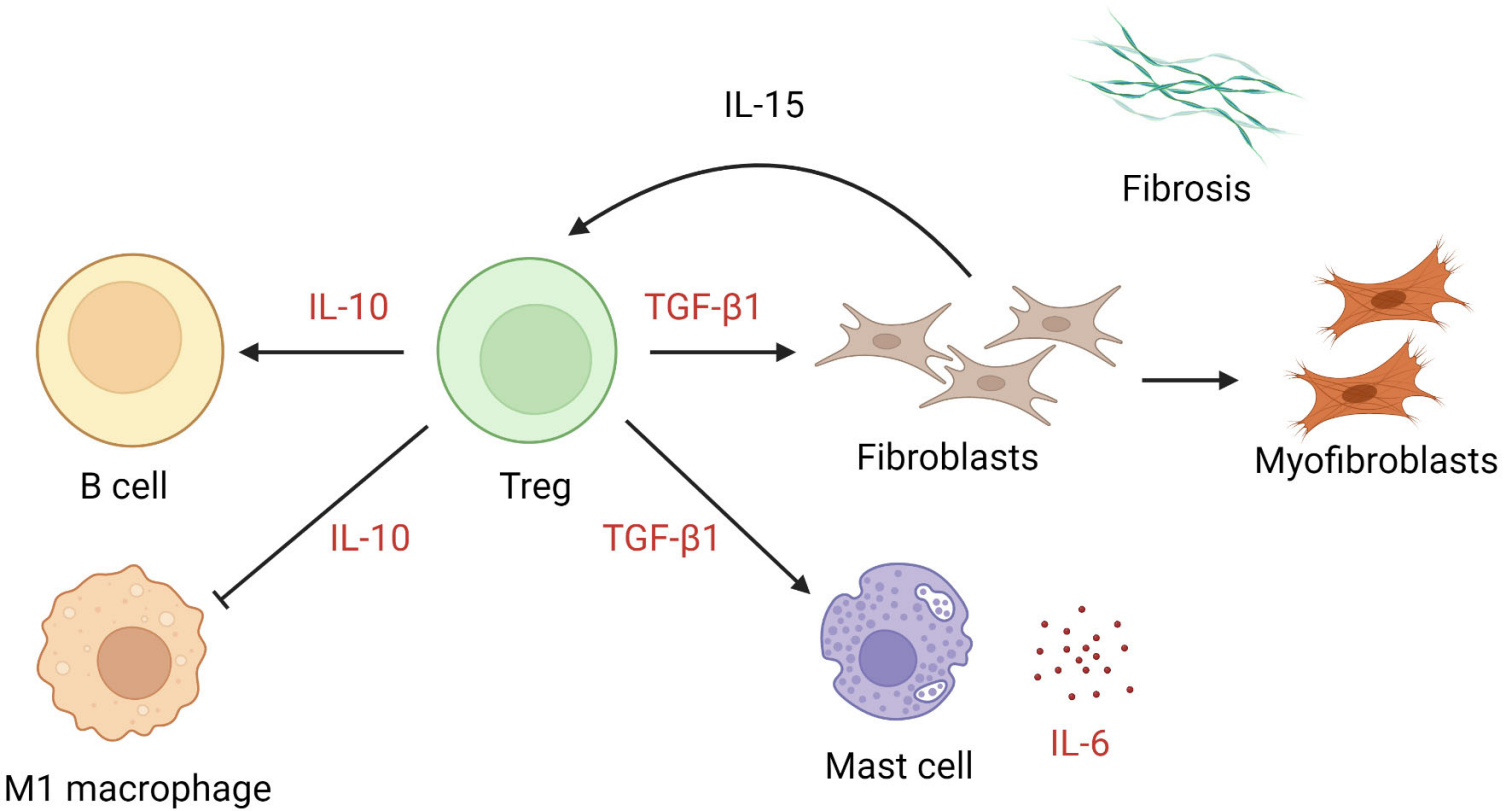
The role of regulatory T cells in keloid pathogenesis. Tregs exert effects through the action of IL-10 and TGF-β1, leading to suppression of M1 macrophages, activation of keloid fibroblasts, and mast cell production of IL-6. *IL*, interleukin; *TGF-β1*, transforming growth factor β1; *Tregs*, regulatory T cells.

The numbers of Tregs are increased in keloid lesions (64, 65). Tregs proliferate after cellular contact with dermal fibroblasts in the presence of IL-15 in chronically inflamed skin (116). In keloids, they promote preferential accumulation of collagen III in the presence of anti-CD3/CD28 (65). In patients with multiple keloid scars, the local infiltration of Tregs was found to be coupled with a reduction in circulating CD4+ CD25high FOXP3+ Tregs (117). Whether the apparent excess of local Tregs is pathogenic or merely represents a response to inflammation remains unclear. TGF-β1 and IL-10 are key cytokines secreted by Tregs and exert an autocrine effect (118, 119). The former mediates elaboration of matrix proteins and stimulates the production of IL-6 by mast cells (120), while the latter downregulates proinflammatory macrophages and promotes B cell activation and immunoglobulin secretion (121) (**Figure 3**). Interestingly, IL-10, rather than IFN-γ, antagonizes the TGF-β1 effect on keloid fibroblasts (93, 122). In muscle, Tregs are known to accumulate at injured sites and modulate the polarization of M1 macrophage to M2 macrophage (123). It is likely that they assume a similar coordinating role in wound healing. Further investigations are required to determine the extent to which Tregs alter the balance between M1 and M2 macrophages and contribute to keloidogenesis.

### 2.4 Chronic stimulation and exhaustion of CD8+ T cells

In normal skin, the majority of T cells are CD45RO+ memory T cells. The same holds true in keloids, with a significantly higher proportion of effector memory CD8+ T cells (T_EM_) and CD103+CD8+ resident memory T cells (T_RM_) (117). T_RM_ are known to trigger an exaggerated inflammatory response to stimuli (124). Keloid memory T cells are less adept at producing TNF-α and more prone to generating IFN-γ (117). FOXP3+ CD8- memory T cells are also defective with decreased IL-10 secretion, resulting in exuberant but dysregulated T cell responses in keloids (117). Further adding to the dysregulation, the expression of granzyme B+ CD8+ cytotoxic T cell is downregulated in keloids, a feature presumably related to the characteristic uncontrolled growth. A recent single-cell RNA study discovered that chronic antigenic stimulation in keloids result in enhanced surface NKG2A expression on CD8+ T cells and natural killer (NK) cells (125, 126), with resultant suppression of cytotoxic T cells *via* the NKG2A-soluble human leukocyte antigen-E (sHLA-E) axis (101). IL-15 (127) and TGF-β (128) were implicated in this process. The enhanced expression of the NKG2A/CD94 complex on CD8+ cytotoxic T cells is correlated with progression of keloids. The level of sHLA-E reflects clinical response to intralesional therapy (triamcinolone and 5-fluorouracil) and predicts recurrence risk (101). Furthermore, the degree of sHLA-E elevation could differentiate keloid scars from certain malignant mimics, with the former exhibiting significantly higher levels of sHLA-E (101). Monalizumab, a humanized anti-NKG2A IgG4 monoclonal antibody, exerts an antitumor effect by unleashing both cytotoxic T cells and NK cells (125). The agent has been tested in clinical trials as part of the immunotherapeutic regimens for advanced solid organ cancers, such as recurrent/metastatic squamous cell carcinoma of the head and neck (129), unresectable stage III non-small-cell lung cancer (130), and recurrent gynecologic malignancies (131). Further studies are required to determine the therapeutic potential of NKG2A/CD94 blockade for keloids.

### 2.5 Dendritic cells

Dermal infiltration of factor XIIIa (FXIIIa)-positive DCs is increased in keloid scars comparing to hypertrophic scars and mature scars (132, 133). These potent antigen-presenting cells are thought to take part in the pathogenic epidermal–dermal interactions in keloids (132), and DC-derived TGF-β could contribute to the differentiation of Tregs. RNA sequencing study confirmed increase of DC markers CD80 and CD86, as well as markers typical of atopic DCs (OX40L+, FCϵR1+) in both lesional and nonlesional skin of keloid patients (85). Unlike in atopic dermatitis, where DCs have been linked to mast cell activation and Th2, Th17 and Th22 differentiation (134, 135), the exact action of DCs in keloids is less clear.

### 2.6 Natural killer cells

Flow cytometric analyses of keloid single-cell suspensions have shown an unusually high number of NK cells (79). Although their role in keloidogenesis is less well described, NK cells express the surface NKG2A/CD94 complex and thus are implicated in the NKG2A-sHLA-E axis (101). Therefore, it is possible that NK cell activity is relatively suppressed in the TGF-β-rich, chronically inflamed keloid milieu, and that a phenomenon paralleling uncontrolled cancerous growth due to NK and cytotoxic T cell exhaustion is likely present.

## 3 Key cytokine pathways in keloid formation

**Figure 4:**
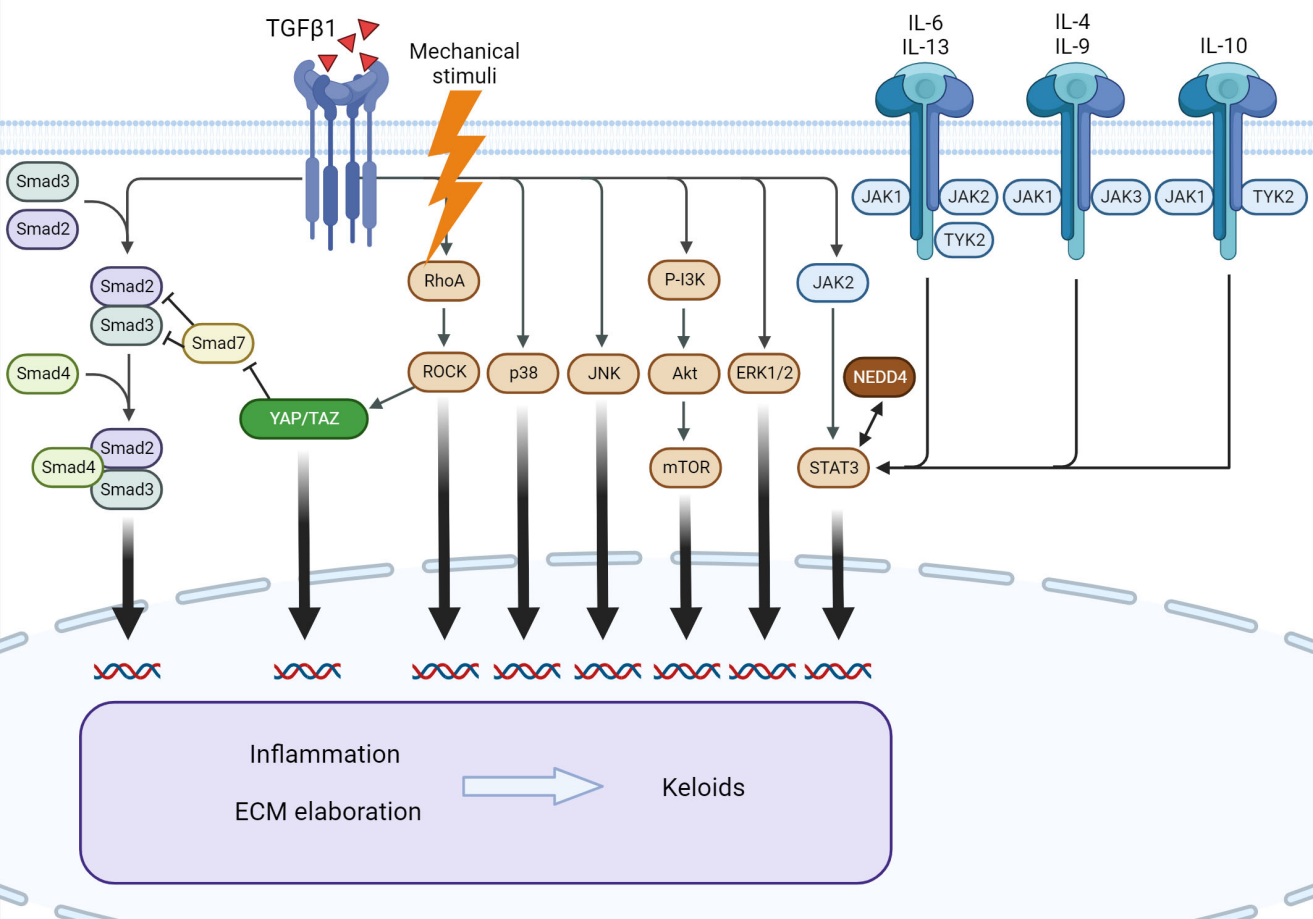
Signaling pathways involved in keloid formation. Several downstream pathways of TGF-β1 participate in keloid pathogenesis. Mechanical stimuli exert an effect through Rho/ROCK and YAP/TAZ, leading to the modulation of canonical TGF-β1 signaling. The interleukin family of cytokines and NEDD4 exert profibrotic action *via* STAT3, independent of TGF-β1. *ERK*, extracellular signal-regulated kinase; *IL*, interleukin; *JAK*, Janus kinase; *JNK*, c-Jun N-terminal kinase; *mTOR*, mammalian target of rapamycin; *NEDD4*, neural precursor cell expressed, developmentally downregulated 4; *PI3K*, phosphatidylinositol-3-kinase; *ROCK*, RhoA/Rho-associated protein kinase; *STAT3*, signal transducer and activator of transcription 3; *TGF-β1*, transforming growth factor β1; *TYK2*, tyrosine kinase 2; *YAP/TAZ*, Yes-associated protein/transcriptional coactivator with PDZ-binding motif.

Keloids are characterized by dysregulation of multiple signaling pathways and associated cytokines. The best described are IL-6/IL-17, IL-4/IL-13, canonical and non-canonical TGF-β1, and JAK/STAT signaling (**Figure 4**).

### 3.1 The essential role of IL-6 in inflammation

IL-6 signals through the JAK1–signal transducer and activator of transcription (STAT) 3 pathway and the extracellular signal-regulated kinase (ERK) 1/2–mitogen-activated protein kinase (MAPK) pathway. Both pathways have been implicated in keloid ECM gene expression and collagen synthesis (90, 92). IL‐6 and the soluble IL‐6 receptor (sIL-6R) are essential for collagen production (136). Similar to patients with systemic sclerosis (137), patients with keloids show elevated IL-6 in the serum and skin (38, 55, 90). In addition, IL-6 polymorphisms have been associated with susceptibility to keloid formation across populations (55–57).

IL-6 is pivotal to the transition from acute to chronic inflammation via initiation of a profibrotic state (138–140). Specifically, the cytokine modulates the fibrogenic crosstalk between fibroblasts and keratinocytes by inducing proinflammatory cytokines (IL-1β and TNF-α) in monocytes *via* MAPK and nuclear factor kappa-light-chain-enhancer of activated B cell (NF-κB) signaling (141). Keratinocyte growth factor production by fibroblasts is enhanced, and the activated keratinocytes in turn produce oncostatin M, triggering STAT3 signaling in dermal fibroblasts (141). IL-6 production is increased in response to enhanced TGF-β1 signaling as a downstream effector (via PI3K and p38-MAPK) (142), and in turn it enhances TGF-β1 production by macrophages (64), creating a positive feedback loop. IL-6 is also crucial to Th2 and M2 macrophage polarization by initiating IL-4 secretion by CD4+ T cells) and upregulating IL-4 receptors (IL-4R) on macrophages (141). Of note, several experimental therapies for keloids and other forms of cutaneous fibrosis directly or indirectly antagonize IL-6. Examples include corticosteroids, verapamil, angiotensin receptor blocker/angiotensin converting enzyme inhibitors, tocilizumab, pirfenidone, and ultraviolet A (87, 141). TNF-α-stimulated gene-6 (TSG-6), a protein suppressed in keloid fibroblasts, has been shown to attenuate IL-1β, IL-6, and TNF-α when intradermally injected into hypertrophic scars (143). The IL-17/IL-6 axis is crucial to sustaining a cytokine-rich, chronically inflamed niche, augmented by an autocrine loop with increased differentiation of Th17 and subsequent heightened secretion of IL-6 (38). IL-17-mediated enhancement of stromal cell-derived factor-1 (SDF-1) in keloid fibroblasts further reinforces Th17 differentiation *via* STAT3 mediation (89). This hyperinflammatory milieu is the most prominent perilesionally (89). Through upregulation of hypoxia-inducible factor-1α (HIF-1α) and STAT3, IL-17 impairs autophagy of both normal and keloid fibroblasts, resulting in increased necroptosis and fibrosis. Antagonization of IL-17 *via* HIF-1α or SDF-1α suppression has been demonstrated *in vitro* (48, 89).

### 3.2 The role of type 2 immunity: IL-4/IL-13

Several studies have investigated the association between keloids and other conditions characterized by the Th2 response. The results are variable, with some studies reporting a positive correlation with atopic dermatitis (6, 12, 13, 81). Th2 immunity is involved in normal wound healing as well as various fibrotic conditions (144). IL-4 and IL-13 are key Th2 cytokines that have wide-ranging influence across cell types as their receptors are commonly present (144). The binding of IL-4 and IL-13 to their cognate receptors activates the IL-4Rα/STAT6 signaling pathway, a TGF-β-independent profibrotic mechanism (145). Both IL-4 and IL-13 independently participate in normal and pathogenic healing. Topical IL-4 significantly accelerates the rate of fibrotic tissue formation, whereas IL-4 antisense oligonucleotides attenuate the healing process in animal models (146). In mouse models of systemic sclerosis, anti-IL-4 monoclonal antibodies prevent progression of cutaneous fibrosis by reducing dermal collagen deposition (147). On the other hand, IL-13 has been shown to directly contribute to fibroblast proliferation and differentiation. IL-13 enhances the expression of type I collagen, α-smooth muscle actin (α-SMA), and other essential proteins involved in fibrogenesis. Furthermore, tissue inhibitors of metalloproteinases are attenuated while matrix metalloproteinases are upregulated in keloid fibroblasts treated with IL-13 (83).

The expression of IL-4, IL-13 and their respective receptors is enhanced in keloid scars (81–83, 85, 144, 145). Regression of chronic keloids has been achieved with Th2-targeting therapy with dupilumab (an anti-IL-4Rα agent) in a case report (81). Others showed variable efficacy (148, 149). Molecular profiling of keloids with RNA sequencing demonstrated a significant increase in Th2 expression in both lesional and non-lesional skin of keloid patients (85). The relative dominance of the Th2 response has been attributed to the anti-apoptotic effect conferred to CD4+ T cells by IL-4 (39). IL−4− and IL−13−activated macrophages (M2 macrophages) are critical to resolving inflammation during wound repair (150, 151). In chronic inflammation, these cytokines have been shown to upregulate miR-142-5p and suppress miR-130a-3p in macrophages, leading to a sustained profibrogenic phenotype (152). Human dermal fibroblasts treated with IL-4 and IL-13 exhibit drastically elevated levels of periostin mRNA with enhanced secretion (82). Periostin is an important promoter of RhoA/Rho-associated protein kinase (ROCK) pathway-mediated TGF-β1 secretion impliacted in pathological scarring (82, 153). In systemic sclerosis, periostin is correlated with skin disease severity (154), and the serum level of IL-13 recflects severity of both skin fibrosis (155). The clinical utility of these biomarkers in keloids remains to be determined.

### 3.3 The pivotal role of TGF-β1

TGF-β1 is one of the most studied mediators of fibrosis. It is frequently implicated in keloid pathogenesis. TGF-β1 has a wide range of cellular sources, including fibroblasts, monocytes, T cells, and platelets (156). As a key regulator of fibrogenesis, this pleiotropic cytokine plays a pivotal role in various cutaneous and solid organ fibrotic disorders, as well as in tumorigenesis *via* induction of cancer-associated fibroblasts (156). Several monoclonal antibodies, small molecule inhibitors, small interfering RNAs (siRNAs), and antisense oligodeoxynucleotides targeting TGF-β1 signaling are currently under development (156).

The induction by TGF-β1 of various growth factors, including connective tissue growth factor (CTGF) and VEGF, is crucial to the maintenance of the ECM. Moreover, TGF-β1 exerts an autocrinal effect that downregulates dipeptidyl peptidase-4 (DPP4) expression, contributing to a chronically inflamed state with elevated levels of the extracellular C-X-C motif chemokine ligand 12 (CXCL12) (157). Fibroblasts in keloids are considerably sensitive to TGF-β compared to those in hypertrophic scars (158, 159). These abnormal fibroblasts are able to overcome Fas-mediated apoptosis when augmented by TGF-β1 (160). TGF-β1-induced smooth muscle actin (SMA) expression in keloid fibroblasts contributes to increased cell rigidity, a phenomenon common to both keloids and scleroderma (161). SMA expression is linked to wound contracture, and the process can be inhibited with treatment with recombinant human decorin, TNF-like weak inducer of apoptosis (TWEAK), and SB-431542, a novel specific inhibitor of TGF-β1 receptor kinase (162). TGF-β1 is also capable of upregulating C-MYC and its downstream splicing regulator polypyrimidine tract-binding protein—a key factor in tumorous growth—in keloid fibroblasts (163). Furthermore, altered interaction between TGF-β isoforms at the receptor level in keloid fibroblasts has been described (164–167). The ratio of these isoforms may cause a tendency to fibrosis (168). Accordingly, a novel truncated type II TGF-β receptor has been designed as an anti-scarring agent (169, 170). Both canonical and non-canonical TGF-β1 signaling are implicated in modulating the keloid keratinocytes to possess a metabolic profile similar to those undergoing epithelial–mesenchymal transition with increased invasiveness (45, 171–173).

#### 3.3.1 Modulators of the TGF-β1/Smad pathway

The canonical TGF-β signaling is enhanced in keloids, and strategic targeting of TGF-β1/Smad has been shown to retard keloid fibroblasts *in vitro* or in animal models (**Supplemental Table 1**). Upstream modulators of the TGF-β1/Smad pathway, including activating transcription factor 3 (174), CR6-interacting factor 1 (175), NLR family CARD domain containing 5 (NLRC5) (176), and nuclear receptor subfamily 3, group C, member 1 (NR3C1), are overexpressed in keloid fibroblasts. HIF-1α and high temperature requirement factor A1 activate the TGF-β1/Smad pathway and promote keloid formation (46, 177). S100A4, a small, calcium-binding protein involved in skin and solid organ fibrosis, is upregulated in keloid fibroblasts and inhibited by calcimycin (178). Syndecan-1, a cell surface proteoglycan highly expressed in wounds, also enhances the pathway in keloids (179). Post-translational sumoylation amplifies TGF-β1/Smad signal transduction in keloids (47).

MicroRNAs are small regulatory RNAs capable of altering post-translational gene expression. In keloid fibroblasts, the anti-fibrotic regulators miR-200c (180), miR-92b (181), miR-1224-5p (182), and miR-133a-3p (183) are expressed at low levels and pro-fibrotic miR-21is overexpressed, altering the activity of the TGF-β1/Smad pathway (184). Peroxisome proliferator-activated receptor-γ agonists have been shown to induce miR-92b expression and thus lower TGF-β1 expression in keloids (181). MicroRNA expression is modulated by long-noncoding RNAs. In keloids, fibroblast behavior is altered in the presence of different long-noncoding RNAs (180, 185–188). For example, LINC01116 contributes to a pro-fibrotic state in keloid tissue *via* editing of miR-3141 (185). In addition, the BMP and activin membrane-bound inhibitor (189), Dickkopf-3 (190), and the receptor for activated C-kinase 1 (191) attenuate TGF-β1-induced fibrosis; all are downregulated in keloid fibroblasts. Smad-7 provides negative feedback to the TGF-β1/Smad system. The molecule is suppressed due to a marked increase in the level of TGF-β inducible early gene-1 in keloids (192). Downregulation of TRAF3IP2 in keloid fibroblasts by FOXO4 attenuates the growth of keloid scars (193). IL-37 is a broad inhibitor of innate inflammation and regulator of TGF-β (194, 195). Recent studies have uncovered its role in modulating several metabolic pathways and a potential role in reversing trained immunity (196). As seen in idiopathic pulmonary fibrosis (197), lower serum levels of IL-37 were found to indicate higher keloid severity (95).

#### 3.3.2 Non-canonical TGF-β pathways

Several non-canonical TGF-β pathways are involved in keloid formation. These include the MAPK (94, 198, 199), ERK 1/2 (44, 200) phosphatidylinositol-3-kinase (P-I3K)/AKT (44, 104, 200–202), c-Jun amino-terminal kinase (94), p38 mitogen-activated protein kinase (p38/MAPK) (94, 203–205), and Rho-like (82) signaling pathways. The multi-kinase inhibitor sorafenib induces cell arrest of keloid fibroblasts by blockade of the intracellular TGF-β/Smad and MAPK/ERK pathways (206). JUN (an oncogene encoding the c-Jun protein) initiates fibrosis *via* CD36 in both human and murine hypertrophic scar fibroblasts, and the blockade of CD36 exerted an anti-scarring effect in the murine model (207).

#### 3.3.3 Bridge to mechanical transduction: Reciprocal cross-regulation with the integrin and Yes-associated protein/transcriptional coactivator with PDZ-binding motif pathways

Crosstalk between TGF-β and mechanical transduction pathways is increasingly recognized. Among these pathways, the integrin pathway (79, 142) and the Hippo/Yes-associated protein/transcriptional coactivator with PDZ-binding motif (YAP/TAZ) pathway (43, 208, 209) are the most recognized. In addition, TGF-β interacts with Wnt/β-catenin activity in dermal fibroblasts, upregulating ECM genes (210, 211). YAP/TAZ are important actors in cellular mechanical transduction. These transcriptional factors are regulated mostly by cell–cell adhesion and cell–ECM attachment *via* integrins (212). Conditions that cause stiffening of the ECM, such as inflammation, lead to a lower threshold of YAP/TAZ activation (212). IL-6 is also known to activate YAP through gp130 signaling (212). Activated YAP/TAZ translocate into keloid fibroblast nuclei, a step required for wound healing (208). In liver cirrhosis, YAP/TAZ contribute to tissue fibrosis *via* enhanced SMA expression, promoting the transformation of fibroblasts into myofibroblasts. YAP/TAZ are also implicated in the sustained profibrotic transcriptional profile of idiopathic pulmonary fibrosis (213). Targeted knockdown of YAP or TAZ has been shown to significantly inhibit the activity and induce apoptosis of keloid fibroblasts (208). Inhibition of Rho/Rho kinase signaling, a major upstream regulator of YAP/TAZ, also attenuates fibroblast activity (82, 214). Manipulation of the YAP/TAZ-associated pathways could potentially reduce keloid scarring. A recent study identified a subpopulation of dermal Engrailed-1 lineage-negative fibroblasts in cell transplantation and transgenic mouse models that could give rise to scar-forming Engrailed-1 lineage-positive fibroblasts during adult wound healing (215). The process is initiated by canonical mechanotransduction signaling and depends on YAP (215). Inhibition or knockout of YAP prohibits Engrailed-1 activation, favoring scarless (regenerative) wound healing *via* Engrailed-1 lineage-negative fibroblasts (215). Verteporfin, a small-molecule YAP inhibitor, has been proposed as a potential novel agent for promoting regenerative skin healing without compromising the healing process (216).

### 3.4 Janus kinase/signal transducers and activators of the transcription pathway

STAT3 is highly expressed and phosphorylated in keloid tissue with increased activation of JAK2 (217). Moreover, STAT3 activity is correlated with fibroblast proliferation and migration, as well as collagen deposition, mainly due to dysregulated secretion of cytokines resulting from altered epithelial–mesenchymal interactions (218). Attenuation of such activity can be achieved with JAK2/STAT3 inhibitors or STAT3 siRNA (217, 219). Cytokines enriched in the keloid microenvironment, especially IL-6 and OSM, are strong activators of the JAK/STAT system. Various Th2- and Th17-cytokines, including IL-4, IL-10, IL-13, and IL-17, also signal through JAK/STAT (85, 87). IL-6-specific hyperactivation of STAT3 has been shown to be profibrotic due to the induction of Gremlin (a bone morphogenetic protein [BMP] antagonist), which in turn sustains canonical TGF-β signaling (136). Recently, RNA sequencing analyses confirmed robust expression of JAK3 in keloid tissue (85), and positioned STAT3 in a feedforward loop regulating a myriad of downstream target genes involved in keloidogenesis (220). From a metabolic viewpoint, keloids exhibit accelerated glycolysis reminiscent of Warburg metabolism, a unique adaptive state presumably induced by JAK2/STAT3 (50, 221). Interestingly, an *in vivo* study demonstrated regulation of keloid fibroblast activity at the cost of a worsened hyperglycolytic state with JAK1/2 blockade (222). Epigallocatechin-3-gallate (EGCG), a green tea extract, has been found to possess chemopreventive properties, including suppression of STAT3 signaling, potentially inhibiting keloid growth (219). ASC-J9, an inhibitor of STAT3 phosphorylation, has shown efficacy in suppressing keloid fibroblasts (223). AG490, a selective JAK2/STAT3 inhibitor, and STAT3−specific decoy oligodeoxynucleotides are also beneficial *in vitro* (224). Oral small-molecule JAK inhibitors are effective in treating skin and pulmonary diseases of systemic sclerosis (225). In a case report, tofacitinib, a pan-JAK inhibitor, facilitated control of keloid scar (226).

STAT3 was recently discovered as a transcription factor for the neural precursor cell expressed, developmentally downregulated 4 (NEDD4) gene (227). NEDD4 encodes a ubiquitin ligase involved in protein degradation and has been associated with susceptibility to keloids (30, 32, 33, 228–231). NEDD4 transcript variant 3 is overexpressed in keloid skin and is responsible for heightened activation of NF-κB *via* interaction with receptor interacting protein, an adaptor protein (29). NF-kB is more prominent in keloids than in normal skin and contributes to impaired apoptosis of fibroblasts (37, 232). Aspirin may potentially prevent this effect (232). NEDD4 regulates cell contact inhibition and T cell factor/β-catenin transcriptional activity (231). It is also linked to fibronectin and type 1 collagen expression (231). A positive feedback loop between STAT3 and NEDD4 has been described (29), and silencing of NEDD4 also attenuates STAT3 (29, 227), making NEDD4 a potential therapeutic target in keloids.

## 4 Other potential therapeutics

Fibroblast activation protein (FAP), a membrane-bound enzyme with structural similarity to DPP4, is found almost exclusively on activated fibroblasts and myofibroblasts under pathological conditions (233), making it a potential target for selective inhibition. Similar to DPP4, FAP upregulates extracellular CXCL12 (234). In addition to its enzymatic activity against the ECM (and thus its association with lesion invasiveness (234, 235), the molecule is likely pluripotent with immunomodulatory properties (234). The FAP expression level is enhanced in keloid fibroblasts (234, 235) and FAP modulation has been shown to attenuate the invasiveness of scars (235). As a marker of pathological fibroblast activation, FAP is a novel subject of interest in solid tumors and connective tissue disorders. Previous studies have shown that FAP chimeric antigen receptor-T cell therapy may be limited by systemic toxicity as FAP is also expressed on multipotent bone marrow stromal cells (236, 237). On the other hand, FAP-inhibiting radiopharmaceuticals have shown theranostic promise in various malignancies and other disorders characterized by tissue fibrosis, such as systemic sclerosis (238), rheumatoid arthritis (239), and IgG4-related disease (240). Targeted photodynamic therapy with an anti-FAP photosensitizer exhibits a dose-dependent therapeutic effect on skin fibroblasts of patients with systemic sclerosis (238).

Additional pathway abnormalities, such as Notch and Toll-like receptor signaling pathways, have been implicated in keloid pathogenesis (51, 241, 242). Human adipose-, amnion-, bone marrow- and Wharton’s jelly-derived mesenchymal stem cells have been shown to inhibit proliferation, migration, and synthesis of keloid fibroblasts *in vitro*, presumably though paracrine effects (243–249). The TGF-β1/Smad and TGF−β2/Smad3 pathways, Notch-1, and cyclooxygenase-2/prostaglandin E2 cascade were all implicated (243–245). Further investigations are warranted to evaluate the *in vivo* effects of these pathways.

## 5 Current challenges and future direction in keloid research

Even with modern technologies, several factors complicate our understanding of keloidogenesis. The lack of an ideal animal model has impeded experimental investigations, and the examination of the nature of keloid scars is limited by sample size. Moreover, the lack of standardization of the site of tissue sampling complicates the interpretation of study results. We previously reported that the inflammatory activity within a keloid scar is most vigorous at the periphery, corresponding to the gradational change in skin tension (31). The gene signatures also varied at the leading edge, center and top of keloid lesions (250). Theoretically, anti-inflammatory measures would be most beneficial at the initial inflammatory stage of wound healing and at the periphery of the scar. Anti-fibrotic therapy, on the other hand, ameliorates the later stages and the more central part of the lesion before scar maturation. To allow for timely and appropriate (i.e., without compromising the healing process) modulation of immune pathways, the mechanisms regulating the transition and spatio-temporal overlap across stages need to be better understood. Studies focusing on explicating the cellular and molecular processes of wound healing, could be of immense value to our understanding and management of keloid disorder.

## 6 Conclusions

The keloid microenvironment is characterized by an exuberant inflammatory response to mechanical and non-mechanical stimuli, resulting in a complex interplay between various hyperactivated immune components with an ultimately profibrotic cytokine profile and signaling. Manipulation of isolated elements or pathways has shown variable efficacy, mostly in an experimental setting. Keloid is increasingly characterized by an inflammatory process, and local treatment might be insufficient for long-term control. Newer biologics and small molecule drugs allow for more specific and systemic targeting of immune pathways. For both approved and experimental drugs, a critical issue is the timing of intervention, as premature suppression of either inflammation or fibrosis could impair wound healing. Further investigations to disentangle the delicate process of wound healing are thus crucial for a more targeted management of keloids.

## Author contributions

Conceptualization, C-CL, C-HT, and C-BC; methodology, C-CL, C-HT, and C-BC; resources, C-HT, W-HC, and C-BC; writing—original draft preparation, C-CL, C-HT, and C-BC; writing—review and editing, C-CL, C-HT, C-HC, Y-CY, W-HC, and C-BC; visualization, C-CL, C-HT, and C-BC; supervision, C-HT, W-HC, and C-BC; project administration, C-HT, C-HC, Y-CY, W-HC, and C-BC. All authors contributed to the article and approved the submitted version.
