# Supplementary material for: An updated review of the immunological mechanisms of keloid scars
Source: Front Immunol. 2023 Mar 22;14:1117630. doi: 10.3389/fimmu.2023.1117630 (PMC10075205; doi:10.3389/fimmu.2023.1117630)
Supplement: Supplementary file 1 [file Table_1.docx]

**Supplemental table 1**. Strategic targeting of the TGF-β1/Smad pathway with potential therapeutic effects on keloids.

| Naturally occurring compounds | - Quercetin (1) - Prostaglandin E2 (PGE2) (2, 3) - Curcuminoids (4) - Asiaticoside (5) - Asiatic acid (6) - Oxymatrine (7) - Compound *Astragalus* and *Salvia miltiorrhiza* extract (CASE) (8) - *Aneilema keisak* extract (9) - Se-ZGTP-I (a selenium-containing polysaccharide from Ziyang green tea) (10) - Aspidin PB (11) - Flavonoids (12) - Ginsenoside Rg3 (13) |
| --- | --- |
| Synthetic agents | - Tamoxifen (14) - Tacrolimus (FK506) (15) - Thalidomide (16) - Troglitazone (17, 18) - Simvastatin (19) - Halofuginone (20) - Metformin (21) - ADP355 (an adiponectin-based peptide) (22) |
| Others | - Cryosurgery (23) - Photodynamic therapy with *Xanthium stramarium* (XAS) and *Psoralea corylifolia* (PSC) (24) - Hypocrellin A photosensitizer and light-emitting diode (LED) red light (25) - Ultraviolet B (26) - Flashlamp pulsed dye laser (27, 28) |

# References

1. Phan TT, Lim IJ, Chan SY, Tan EK, Lee ST, Longaker MT. Suppression of Transforming Growth Factor Beta/Smad Signaling in Keloid-Derived Fibroblasts by Quercetin: Implications for the Treatment of Excessive Scars. *J Trauma* (2004) 57(5):1032-7. doi: 10.1097/01.ta.0000114087.46566.eb.

2. Sandulache VC, Parekh A, Li-Korotky H, Dohar JE, Hebda PA. Prostaglandin E2 Inhibition of Keloid Fibroblast Migration, Contraction, and Transforming Growth Factor (Tgf)-Beta1-Induced Collagen Synthesis. *Wound Repair Regen* (2007) 15(1):122-33. doi: 10.1111/j.1524-475X.2006.00193.x.

3. Hayashi T, Nishihira J, Koyama Y, Sasaki S, Yamamoto Y. Decreased Prostaglandin E2 Production by Inflammatory Cytokine and Lower Expression of Ep2 Receptor Result in Increased Collagen Synthesis in Keloid Fibroblasts. *J Invest Dermatol* (2006) 126(5):990-7. doi: 10.1038/sj.jid.5700227.

4. Hsu YC, Chen MJ, Yu YM, Ko SY, Chang CC. Suppression of Tgf-Β1/Smad Pathway and Extracellular Matrix Production in Primary Keloid Fibroblasts by Curcuminoids: Its Potential Therapeutic Use in the Chemoprevention of Keloid. *Arch Dermatol Res* (2010) 302(10):717-24. Epub 20100818. doi: 10.1007/s00403-010-1075-y.

5. Tang B, Zhu B, Liang Y, Bi L, Hu Z, Chen B, et al. Asiaticoside Suppresses Collagen Expression and Tgf-Β/Smad Signaling through Inducing Smad7 and Inhibiting Tgf-Βri and Tgf-Βrii in Keloid Fibroblasts. *Arch Dermatol Res* (2011) 303(8):563-72. Epub 20110115. doi: 10.1007/s00403-010-1114-8.

6. Bian D, Zhang J, Wu X, Dou Y, Yang Y, Tan Q, et al. Asiatic Acid Isolated from Centella Asiatica Inhibits Tgf-Β1-Induced Collagen Expression in Human Keloid Fibroblasts Via Ppar-Γ Activation. *Int J Biol Sci* (2013) 9(10):1032-42. Epub 20131025. doi: 10.7150/ijbs.7273.

7. Fan DL, Zhao WJ, Wang YX, Han SY, Guo S. Oxymatrine Inhibits Collagen Synthesis in Keloid Fibroblasts Via Inhibition of Transforming Growth Factor-Β1/Smad Signaling Pathway. *Int J Dermatol* (2012) 51(4):463-72. doi: 10.1111/j.1365-4632.2011.05234.x.

8. He S, Yang Y, Liu X, Huang W, Zhang X, Yang S, et al. Compound Astragalus and Salvia Miltiorrhiza Extract Inhibits Cell Proliferation, Invasion and Collagen Synthesis in Keloid Fibroblasts by Mediating Transforming Growth Factor-Β / Smad Pathway. *Br J Dermatol* (2012) 166(3):564-74. Epub 20111205. doi: 10.1111/j.1365-2133.2011.10674.x.

9. Kim WS, Lee JS, Bae GY, Kim JJ, Chin YW, Bahk YY, et al. Extract of Aneilema Keisak Inhibits Transforming Growth Factor-Β-Dependent Signalling by Inducing Smad2 Downregulation in Keloid Fibroblasts. *Exp Dermatol* (2013) 22(1):69-71. doi: 10.1111/exd.12063.

10. Lu L, Chai L, Wang W, Yuan X, Li S, Cao C. A Selenium-Enriched Ziyang Green Tea Polysaccharide Induces Bax-Dependent Mitochondrial Apoptosis and Inhibits Tgf-Β1-Stimulated Collagen Expression in Human Keloid Fibroblasts Via Ng2 Inactivation. *Biol Trace Elem Res* (2017) 176(2):270-7. Epub 20160827. doi: 10.1007/s12011-016-0827-8.

11. Song R, Li G, Li S. Aspidin Pb, a Novel Natural Anti-Fibrotic Compound, Inhibited Fibrogenesis in Tgf-Β1-Stimulated Keloid Fibroblasts Via Pi-3k/Akt and Smad Signaling Pathways. *Chem Biol Interact* (2015) 238:66-73. Epub 20150606. doi: 10.1016/j.cbi.2015.06.005.

12. Zhang Q, Qian D, Tang DD, Liu J, Wang LY, Chen W, et al. Glabridin from Glycyrrhiza Glabra Possesses a Therapeutic Role against Keloid Via Attenuating Pi3k/Akt and Transforming Growth Factor-Β1/Smad Signaling Pathways. *J Agric Food Chem* (2022). Epub 20220825. doi: 10.1021/acs.jafc.2c02045.

13. Tang M, Bian W, Cheng L, Zhang L, Jin R, Wang W, et al. Ginsenoside Rg3 Inhibits Keloid Fibroblast Proliferation, Angiogenesis and Collagen Synthesis in Vitro Via the Tgf‑Β/Smad and Erk Signaling Pathways. *Int J Mol Med* (2018) 41(3):1487-99. Epub 20180104. doi: 10.3892/ijmm.2018.3362.

14. Chau D, Mancoll JS, Lee S, Zhao J, Phillips LG, Gittes GK, et al. Tamoxifen Downregulates Tgf-Beta Production in Keloid Fibroblasts. *Ann Plast Surg* (1998) 40(5):490-3. doi: 10.1097/00000637-199805000-00008.

15. Wu CS, Wu PH, Fang AH, Lan CC. Fk506 Inhibits the Enhancing Effects of Transforming Growth Factor (Tgf)-Β1 on Collagen Expression and Tgf-Β/Smad Signalling in Keloid Fibroblasts: Implication for New Therapeutic Approach. *Br J Dermatol* (2012) 167(3):532-41. Epub 20120719. doi: 10.1111/j.1365-2133.2012.11023.x.

16. Liang CJ, Yen YH, Hung LY, Wang SH, Pu CM, Chien HF, et al. Thalidomide Inhibits Fibronectin Production in Tgf-Β1-Treated Normal and Keloid Fibroblasts Via Inhibition of the P38/Smad3 Pathway. *Biochem Pharmacol* (2013) 85(11):1594-602. Epub 20130313. doi: 10.1016/j.bcp.2013.02.038.

17. Zhang GY, Yi CG, Li X, Ma B, Li ZJ, Chen XL, et al. Troglitazone Suppresses Transforming Growth Factor-Beta1-Induced Collagen Type I Expression in Keloid Fibroblasts. *Br J Dermatol* (2009) 160(4):762-70. Epub 20081202. doi: 10.1111/j.1365-2133.2008.08989.x.

18. Zhu HY, Bai WD, Li J, Tao K, Wang HT, Yang XK, et al. Peroxisome Proliferator-Activated Receptor-Γ Agonist Troglitazone Suppresses Transforming Growth Factor-Β1 Signalling through Mir-92b Upregulation-Inhibited Axl Expression in Human Keloid Fibroblasts in Vitro. *Am J Transl Res* (2016) 8(8):3460-70. Epub 20160815.

19. Mun JH, Kim YM, Kim BS, Kim JH, Kim MB, Ko HC. Simvastatin Inhibits Transforming Growth Factor-Β1-Induced Expression of Type I Collagen, Ctgf, and Α-Sma in Keloid Fibroblasts. *Wound Repair Regen* (2014) 22(1):125-33. Epub 20131213. doi: 10.1111/wrr.12136.

20. Marty P, Chatelain B, Lihoreau T, Tissot M, Dirand Z, Humbert P, et al. Halofuginone Regulates Keloid Fibroblast Fibrotic Response to Tgf-Β Induction. *Biomed Pharmacother* (2021) 135:111182. Epub 20210201. doi: 10.1016/j.biopha.2020.111182.

21. Jeon HB, Roh H, Ahn HM, Lee JH, Yun CO, Roh TS, et al. Metformin Inhibits Transforming Growth Factor Β-Induced Fibrogenic Response of Human Dermal Fibroblasts and Suppresses Fibrosis in Keloid Spheroids. *Ann Plast Surg* (2021) 86(4):406-11. doi: 10.1097/sap.0000000000002574.

22. Darmawan CC, Montenegro SE, Jo G, Kusumaningrum N, Lee SH, Chung JH, et al. Adiponectin-Based Peptide (Adp355) Inhibits Transforming Growth Factor-Β1-Induced Fibrosis in Keloids. *Int J Mol Sci* (2020) 21(8). Epub 20200418. doi: 10.3390/ijms21082833.

23. Awad SM, Ismail SA, Sayed DS, Refaiy AE, Makboul R. Suppression of Transforming Growth Factor-Beta1 Expression in Keloids after Cryosurgery. *Cryobiology* (2017) 75:151-3. Epub 20170310. doi: 10.1016/j.cryobiol.2017.03.004.

24. Park SY, Park JY, Kim CH, Kang SU, Kim JH, Bark KM, et al. Effects of Xanthium Stramarium and Psoralea Corylifolia Extracts Combined with Uva1 Irradiation on the Cell Proliferation and Tgf-Β1 Expression of Keloid Fibroblasts. *Ann Dermatol* (2013) 25(3):304-9. Epub 20130813. doi: 10.5021/ad.2013.25.3.304.

25. Niu T, Tian Y, Shi Y, Guo G, Tong Y, Wang G. Antiﬁbrotic Effects of Hypocrellin a Combined with Led Red Light Irradiation on Keloid Fibroblasts by Counteracting the Tgf-Β/Smad/Autophagy/Apoptosis Signalling Pathway. *Photodiagnosis Photodyn Ther* (2021) 34:102202. Epub 20210205. doi: 10.1016/j.pdpdt.2021.102202.

26. Wirohadidjojo YW, Radiono S, Budiyanto A, Soebono H. Cellular Viability, Collagen Deposition, and Transforming Growth Factor Β1 Production among Ultraviolet B-Irradiated Keloid Fibroblasts. *Aesthetic Plast Surg* (2011) 35(6):1050-5. Epub 20110514. doi: 10.1007/s00266-011-9732-x.

27. Kuo YR, Wu WS, Jeng SF, Wang FS, Huang HC, Lin CZ, et al. Suppressed Tgf-Beta1 Expression Is Correlated with up-Regulation of Matrix Metalloproteinase-13 in Keloid Regression after Flashlamp Pulsed-Dye Laser Treatment. *Lasers Surg Med* (2005) 36(1):38-42. doi: 10.1002/lsm.20104.

28. Kuo YR, Jeng SF, Wang FS, Chen TH, Huang HC, Chang PR, et al. Flashlamp Pulsed Dye Laser (Pdl) Suppression of Keloid Proliferation through Down-Regulation of Tgf-Beta1 Expression and Extracellular Matrix Expression. *Lasers Surg Med* (2004) 34(2):104-8. doi: 10.1002/lsm.10206.
